# Supplementary material for: Effect of Fluoroquinolone Use in Primary Care on the Development and Gradual Decay of Escherichia coli Resistance to Fluoroquinolones: A Matched Case-Control Study
Source: Antibiotics (Basel). 2022 Jun 18;11(6):822. doi: 10.3390/antibiotics11060822 (PMC9219874; doi:10.3390/antibiotics11060822)
Supplement: Supplementary file 1 [file antibiotics-11-00822-s001.zip › antibiotics-1774995-supplementary.pdf]

Supplementary Table S1. Codes for exposure

ATC	3GC
J01MA02	Ciprofloxacin
J01MA06	Norfloxacin
J01MA12	Levofloxacin

Abbreviations. ATC = Anatomical therapeutic class; FQs = fluoroquinolones

Supplementary Table S2. Codes for considered comorbidities

Comorbidity	ATC	ICD-9-CM
Cancer	L01, L02A, L02B	140, 208
Diabetes	A10	250
COPD	R03BB	490, 491.1, 491.20, 491.21, 491.22, 491.8, 491.9, 492.0, 492.8, 493.22, 496
AIDS	J05A	42
Immunosuppression		279 (except 279.4)
Haemodialysis		399.5, 549.8, 392.7

Abbreviations. ATC = Anatomical therapeutic class; ICD-9-CM = International Classification of Diseases, Ninth Revision, Clinical Modification, AIDS = Acute immune deficiency syndrome; COPD = Chronic obstructive pulmonary disorder.

Supplementary Table S3. Sensitivity analysis performed excluding all patients who received at least one prescription of antibiotics in the 15 days preceding the ID

Variable		Cases N=375	Controls N=917	Adjusted OR (95% CI)	p
**FQ prescriptions in previous year (%)**	**0**	248 (66.13)	785 (85.61)	*Ref.*	*Ref.*
	**1**	61 (16.27)	72 (7.85)	**2.50 (1.68 – 3.72)**	**<0.0001**
	**2**	31 (8.27)	34 (3.71)	**2.46 (1.45 - 4.17)**	**0.001**
	**3+**	35 (9.33)	26 (2.84)	**3.75 (2.09 – 6.74)**	**<0.0001**
At least one other J01 prescription in previous year (%)		174 (46.40)	306 (33.37)	1.19 (0.90-1.56)	0.221
Age, Median (IQ)		78 (68-86)	75 (59-84)	1.11[a] (1.03 – 1.96)	0.007
Gender, Male (%)		253 (61.86)	399 (42.77)	1.45 (1.09 – 1.91)	0.010
Hospitalization days, Median (IQ)		47 (12-116)	9 (0-90)	1.05[b] (1.04-1.07)	<0.0001
Diagnosis of chronic diseases (%)	Diabetes	100 (26.67)	145 (15.81)	1.41 (1.02 – 1.94)	0.032
	COPD	148 (39.47)	236 (25.74)	1.49 (1.12 – 1.99)	<0.0001

[a]OR calculated for 10-year increments; [b]OR calculated for 10-day increments

Abbreviations. ID = index date; FQ = fluoroquinolone; IQ = interquartile range; COPD = chronic obstructive pulmonary disease; QREC = Quinolone-Resistant *E. coli*

Supplementary Table S4. Sensitivity analysis performed excluding all patients with a bacterial culture performed after 48 hours of hospital admission

Variable		Cases N=135	Controls N=287	Adjusted OR (95% CI)	p
**FQ prescriptions in previous year (%)**	**0**	87 (64.44)	232 (81.12)	*Ref.*	*Ref.*
	**1**	20 (14.81)	38 (13.29)	**0.48 (0.15 – 1.37)**	**0.159**
	**2**	15 (11.11)	8 (2.80)	**2.30 (0.34 – 15.76)**	**0.395**
	**3+**	13 (9.63)	8 (2.80)	**18.78 (1.69 – 208.77)**	**0.017**
At least one other J01 prescription in previous year (%)		68 (50.37)	107 (37.41)	0.80 (0.35-1.86)	0.613
Age, Median (IQ)		81 (71-86)	75 (59-85)	1.13[a] (0.87 – 1.48)	0.353

Gender, Male (%)		44 (38.94)	69 (61.06)	2.47 (0.78 – 7.84)	0.126
Number of hospitalizations, Median (IQ)		5 (2-9)	2 (0-4)	1.18 (1.06-1.32)	0.002
Diagnosis of chronic diseases (%)	Diabetes	39 (28.89)	47 (16.43)	2.11 (0.76 – 5.82)	0.150
	COPD	63 (46.67)	76 (26.57)	1.94 (0.71 – 5.28)	0.196

[a]OR calculated for 10-year increments

Abbreviations. ID = index date; FQ = fluoroquinolone; IQ = interquartile range; COPD = chronic obstructive pulmonary disease; QREC = Quinolone-Resistant *E. coli*
